# Homoeolog expression bias and expression level dominance (ELD) in four tissues of natural allotetraploid *Brassica napus*

**DOI:** 10.1186/s12864-020-6747-1

**Published:** 2020-04-29

**Authors:** Mengdi Li, Ruihua Wang, Xiaoming Wu, Jianbo Wang

**Affiliations:** 1 State Key Laboratory of Hybrid Rice, College of Life Sciences, Wuhan University, Wuhan, 430072 China; 2 Key Laboratory of Biology and Genetic Improvement of Oil Crops, Ministry of Agriculture, Oil Crops Research Institute of CAAS, Wuhan, 430062 China

**Keywords:** Homoeolog expression bias, Expression level dominance, *Brassica napus*, Natural allotetraploid

## Abstract

**Background:**

Allopolyploidy is widespread in angiosperms, and they can coordinate two or more different genomes through genetic and epigenetic modifications to exhibit stronger vigor and adaptability. To explore the changes in homologous gene expression patterns in the natural allotetraploid *Brassica napus* (A_n_A_n_C_n_C_n_) relative to its two diploid progenitors, *B. rapa* (A_r_A_r_) and *B. oleracea* (C_o_C_o_), after approximately 7500 years of domestication, the global gene pair expression patterns in four major tissues (stems, leaves, flowers and siliques) of these three species were analyzed using an RNA sequencing approach.

**Results:**

The results showed that the ‘transcriptomic shock’ phenomenon was alleviated in natural *B. napus* after approximately 7500 years of natural domestication, and most differentially expressed genes (DEGs) in *B. napus* were downregulated relative to those in its two diploid progenitors. The KEGG analysis indicated that three pathways related to photosynthesis were enriched in both comparison groups (A_n_A_n_C_n_C_n_ vs A_r_A_r_ and A_n_A_n_C_n_C_n_ vs C_o_C_o_), and these pathways were all downregulated in four tissues of *B. napus*. In addition, homoeolog expression bias and expression level dominance (ELD) in *B. napus* were thoroughly studied through analysis of expression levels of 27,609 *B. rapa*-*B. oleracea* orthologous gene pairs. The overwhelming majority of gene pairs (an average of 86.7%) in *B. napus* maintained their expression pattern in two diploid progenitors, and approximately 78.1% of the gene pairs showed expression bias with a preference toward the A subgenome. Overall, an average of 48, 29.7 and 22.3% homologous gene pairs exhibited additive expression, ELD and transgressive expression in *B. napus*, respectively. The ELD bias varies from tissue to tissue; specifically, more gene pairs in stems and siliques showed ELD-A, whereas the opposite was observed in leaves and flowers. More transgressive upregulation, rather than downregulation, was observed in gene pairs of *B. napus*.

**Conclusions:**

In general, these results may provide a comprehensive understanding of the changes in homologous gene expression patterns in natural *B. napus* after approximately 7500 years of evolution and domestication and may enhance our understanding of allopolyploidy.

## Background

Polyploidy is widespread in plants, especially in angiosperms. Even Arabidopsis, which has a relatively small genome, is no exception, while at least three rounds of ancient polyploidization events occurred in its evolutionary history [1, 2]. Polyploidization is considered to be one of the important mechanisms of angiosperm speciation [3–7] and has been and will continue to be an important force in plant evolution [2, 8]. There are two major types of polyploidy in plants, autopolyploidy and allopolyploidy. Autopolyploids consist of multiple sets of identical or similar genomes from intraspecific genome duplication, while allopolyploids are composed of two or more different homoeologous genomes from interspecific or intergeneric hybridization [9]. Both autopolyploids and allopolyploids are very common in nature [10, 11], and many major crops or cash crops are allopolyploids, such as rapeseed (*Brassica napus*), wheat (*Triticum aestivum*), tobacco (*Nicotiana tabacum*) and cotton (*Gossypium hirsutum*). Allopolyploids exhibiting greater vigor and adaptation to various biotic and abiotic stresses is believed to be critical in the differentiation and speciation of plants [12–14].

After hybridization and polyploidization, the ‘genomic shock’ event [15] occurs in newly formed allopolyploids, which leads to changes in their genomes (including genetic and epigenetic changes) and further leads to the reprogramming of transcriptomes, recombinant proteomes, and metabolomes [5]. Specifically, genetic changes include DNA loss, gene conversion, epistasis, homologous recombination, and ectopic recombination; epigenetic changes that may occur at the transcriptional/posttranscriptional levels include histone modification, DNA methylation, small RNA-mediated gene silencing, and transposon suppression/release [9, 12, 14, 16–18]. These changes in new allopolyploid genomes may bring about extensive gene expression changes [12, 19]. In addition, the gene expression pattern of duplicated genes with similar functions may change during the formation of allopolyploids, which takes several typical patterns, including transgressive up−/downregulation, unequal parental contributions, and silencing [9, 14]. These changes in gene expression patterns are of great significance for allopolyploids; for example, these changes may lead to some phenotypic differences between allopolyploids and their parental species and are also important sources of the dominant phenotypes of allopolyploids [9, 17].

*Brassica napus* (A_n_A_n_C_n_C_n_, 2n = 38) is one of the most widely cultivated important oil crops in the world. This crop not only provides edible oil and important nutrients for human beings but also provides protein-rich food for animals [20]. The allotetraploid *B. napus* was formed by natural hybridization and polyploidization of two diploid progenitors, *B. rapa* (A_r_A_r_, 2n = 20) and *B. oleracea* (C_o_C_o_, 2n = 18), approximately 7500 years ago [21]. A recent study showed that A subgenome of *B. napus* might evolve from the ancestor of European turnip, and the C subgenome might evolve from the common ancestor of kohlrabi, cauliflower, broccoli, and Chinese kale [22]. *B. napus* and its two diploid progenitors are a model system for studying the gene expression and genomic changes in the formation of allopolyploids. To date, many genetic and epigenetic changes in the formation of allotetraploid *B. napus* have been studied, including chromosome pairings [23], chromosomal rearrangements [18, 24–28], transposon activation [29], gene expression changes [28, 30–35], alternative splicing pattern changes [36], epigenetic phenomena [28, 37, 38], and protein expression changes [39, 40]. Moreover, only one study has focused on changes in expression level dominance (ELD) and homoeolog expression bias in newly synthesized allotetraploid *B. napus* and its diploid parents [20]. However, similar studies are limited in natural allotetraploid *B. napus* and its diploid progenitors.

High-throughput transcriptome sequencing technology can provide whole-genome gene expression information with low background signals but accurate quantification [41] and makes it possible to distinguish the expression of homologous genes [20, 42]. In recent years, the genomes of *B. napus* [21, 43, 44] and its two diploid progenitors *B. rapa* [45] and *B. oleracea* [46] have been successfully sequenced, providing an unprecedented opportunity to explore the ELD and homologous expression bias of natural allotetraploid *B. napus* and its two diploid progenitors. In this study, we analyzed the transcriptome of four major tissues (stems, leaves, flowers and siliques) in natural allotetraploid *B. napus* and its two diploid progenitors to explore the gene expression characteristics. In addition, the ELD and homoeolog expression bias were investigated thoroughly in natural allotetraploid *B. napus* and its two diploid progenitors. The results of this study provided a new perspective for the expression of duplicate genes (homoeologs) in naturally occurring allotetraploid *B. napus* and helped to characterize the allopolyploidization processes.

## Results

### Transcriptome sequencing and read mapping

**Fig. 1:**
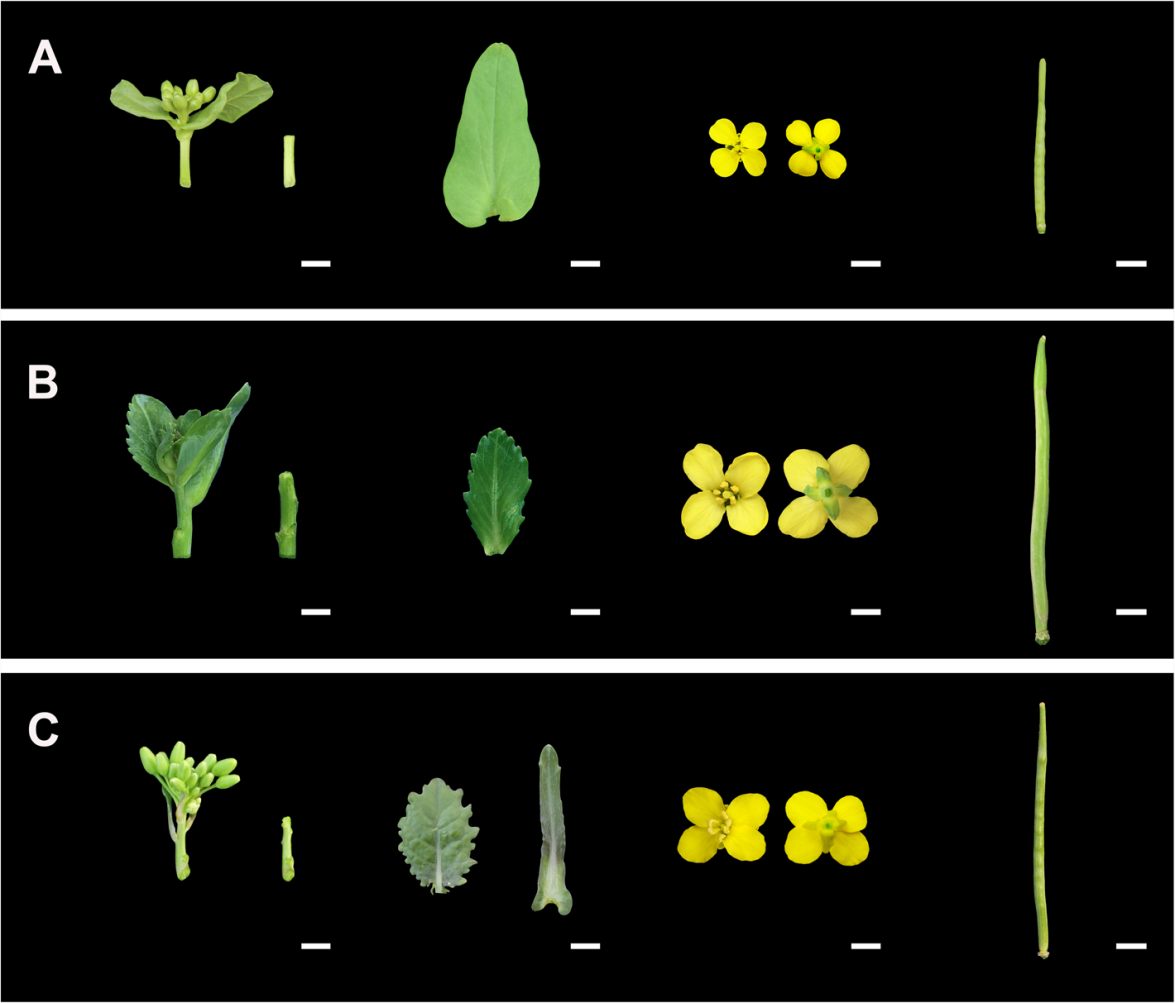
Photos of experimental materials. Inflorescence stems, young leaves, blooming flowers and siliques from *B. rapa* (**a**), *B. oleracea* (**b**) and *B. napus* (**c**)

**Fig. 2:**
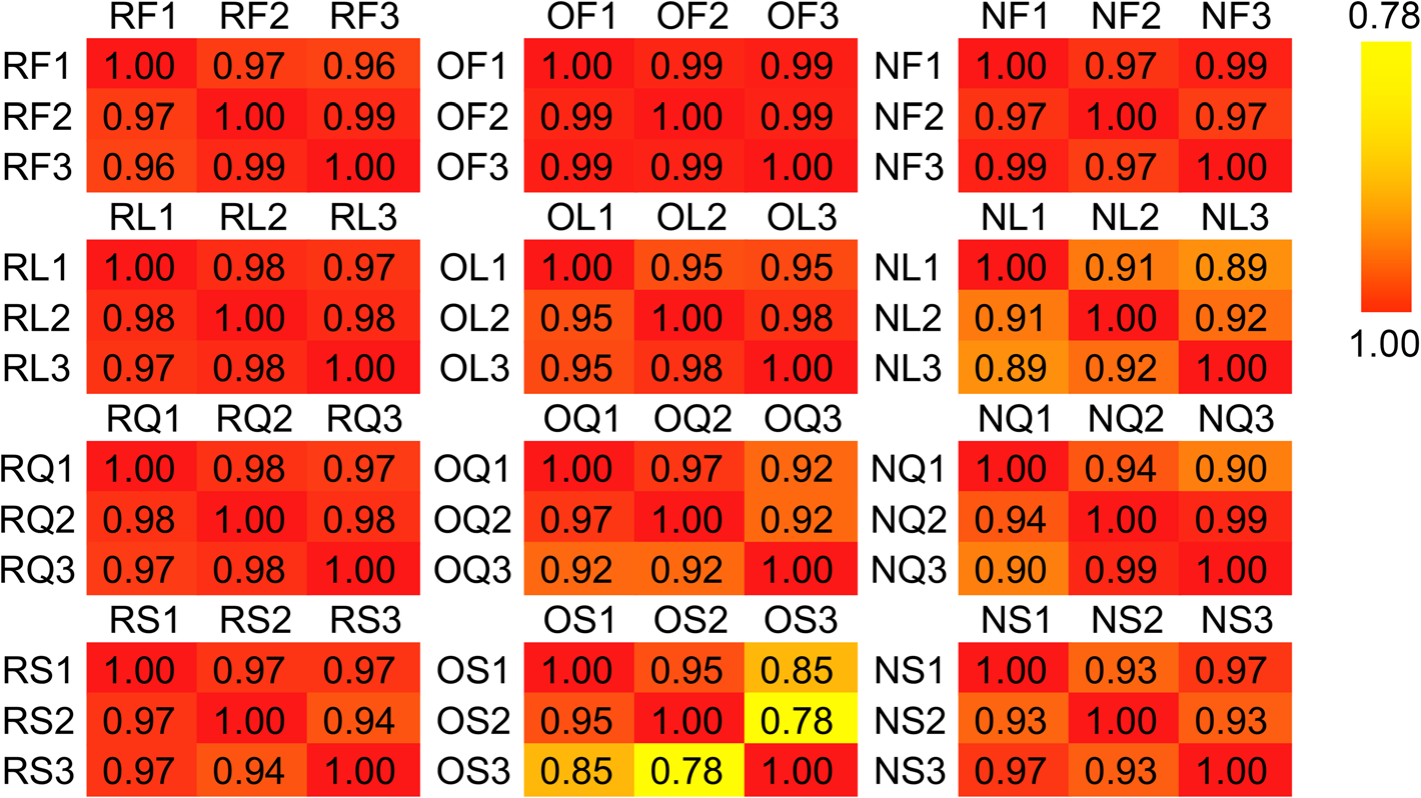
Pearson correlation coefficient between the three biological replicates. The first capital letter represents species (R, O, and N represent *B. rapa*, *B. oleracea*, and *B. napus*, respectively), and the second capital letter represents tissues (S, L, F, and Q represent stems, leaves, flowers, and siliques, respectively). 1, 2 and 3 represent three biological replicates, respectively

High-throughput transcriptome sequencing was used to study and compare the transcript differences in natural allotetraploid *B. napus* relative to its diploid progenitors. The RNA samples from stems, leaves, flowers, and siliques of *B. napus* and its diploid progenitors (Fig. 1) were subjected to paired-end RNA sequencing, each with three biological replicates. After filtering and quality control of the raw reads, a total of 1529.93 million (M) clean reads from 36 RNA libraries were obtained (approximately 42.5 M reads per library, Table 1). The Q30 and Q20 percentage of the reads obtained from all samples exceeded 93.89 and 98.02%, indicating that the sequencing results had high reliability and accuracy (Table 1). An average of 85.5, 63.9, and 63.7% of the reads from the samples of *B. rapa*, *B. oleracea*, and *B. napus* were uniquely mapped to the A genome [47], the C genome [46], and the integrated A-C genome, respectively (Table 1). Gene expression correlations between the three biological replicates were high, and the Pearson correlation coefficient (R) between them mostly exceeded 0.9 (Fig. 2). The transcripts per million reads (TPM) method were used to normalize the gene expression levels, and if the value of TPM was greater than 0, the gene was considered to be expressed in our study. The specific statistics of expressed gene numbers in all samples are shown in Table 2. In total, 41,914, 32,204 and 73,012 genes were detected to be expressed in the four tissues of *B. rapa*, *B. oleracea* and *B. napus*, respectively. Among the 73,012 genes expressed in *B. napus*, 40,831 genes were derived from the A subgenome, and 32,181 genes were derived from the C subgenome.

**Table 1:** Statistics of RNA-seq data for all samples

Species	Tissues	Samples^a^	Total clean reads (M)	Clean reads Q20 (%)	Clean reads Q30 (%)	Uniquely mapping genome ratio (%)
*B. rapa*	Stems	RS1	42.68	99.02	96.94	87.27
		RS2	42.43	99.01	96.91	86.92
		RS3	42.15	99.03	96.96	84.93
	Leaves	RL1	42.51	99.04	97	82.29
		RL2	42.64	98.99	96.89	84.39
		RL3	42.23	98.89	96.69	79.82
	Flowers	RF1	43.00	99.21	97.3	87.85
		RF2	43.09	99.23	97.34	87.87
		RF3	43.12	99.24	97.43	87.77
	Siliques	RQ1	42.08	99.02	96.94	86.61
		RQ2	42.45	99.01	96.91	86.13
		RQ3	42.63	99.03	96.96	84.23
*B. oleracea*	Stems	OS1	43.11	98.02	93.9	63.56
		OS2	42.22	98.08	94.05	65.37
		OS3	42.11	98.14	94.22	63.25
	Leaves	OL1	42.43	98.02	93.89	64.63
		OL2	42.47	98.88	96.26	65.40
		OL3	42.58	98.9	96.29	64.51
	Flowers	OF1	42.09	98.9	96.33	63.96
		OF2	43.24	98.9	96.3	63.26
		OF3	42.94	98.94	96.41	63.38
	Siliques	OQ1	42.93	98.02	93.9	62.90
		OQ2	42.12	98.08	94.05	63.20
		OQ3	42.04	98.14	94.22	63.34
*B. napus*	Stems	NS1	43.01	99.04	96.76	59.63
		NS2	42.57	99	96.63	61.55
		NS3	43.26	99.04	96.75	62.20
	Leaves	NL1	41.60	99.33	97.7	67.86
		NL2	43.15	99.31	97.69	66.16
		NL3	40.66	99.3	97.66	66.47
	Flowers	NF1	43.18	99.05	96.79	64.29
		NF2	42.17	99.04	96.76	64.62
		NF3	40.73	99.02	96.69	66.11
	Siliques	NQ1	42.69	99.04	96.76	62.36
		NQ2	43.07	99	96.63	61.72
		NQ3	42.55	99.04	96.75	61.48

^a^1, 2 and 3 represented three biological replicates

**Table 2:** Statistics of expressed gene numbers in all samples

Samples	Expressed gene numbers on A-genome	Expressed gene numbers on C-genome	Total expressed gene numbers
RF	37,698	–	37,698
RL	37,114	–	37,114
RQ	38,233	–	38,233
RS	35,729	–	35,729
OF	–	29,954	29,954
OL	–	26,728	26,728
OQ	–	30,385	30,385
OS	–	26,793	26,793
NF	36,439	28,813	65,252
NL	33,908	27,057	60,965
NQ	38,290	30,203	68,493
NS	35,933	28,648	64,581

### Differentially expressed genes (DEGs) between *B. napus* and its diploid progenitors

**Fig. 3:**
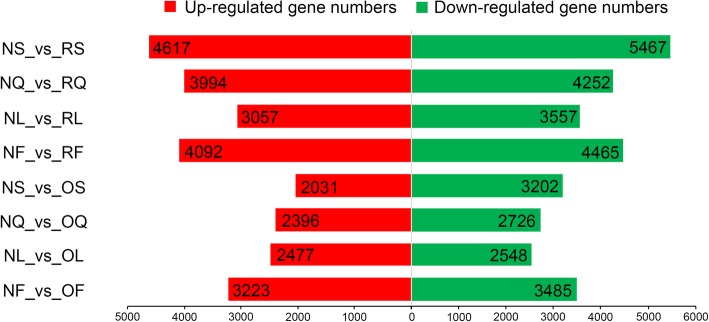
DEGs in four tissues of *B. napus* relative to its two diploid progenitors. The number of upregulated and downregulated genes in each comparison group was represented by red and green bars in the histogram, and the specific number of these genes was recorded in this figure. NS, stems of *B. napus*; NL, leaves of *B. napus*; NF, flowers of *B. napus*; NQ, siliques of *B. napus*; RS, stems of *B. rapa*; RL, leaves of *B. rapa*; RF, flowers of *B. rapa*; RQ, siliques of *B. rapa*; OS, stems of *B. oleracea*; OL, leaves of *B. oleracea*; OF, flowers of *B. oleracea*; OQ, siliques of *B. oleracea*

To study the differences in gene expression between natural allotetraploid *B. napus* (A_n_A_n_C_n_C_n_) and its diploid progenitors (A_r_A_r_ and C_o_C_o_), all DEGs in stems, leaves, flowers, and siliques were identified using DESeq2, with |log_2_ fold change| ≥ 1 and padj ≤0.001. Compared with diploid progenitors *B. rapa*, a total of 17,463 DEGs were identified in four tissues, including 10,084 in stems, 6614 in leaves, 8557 in flowers and 8246 in siliques (Fig. 3). Compared with diploid progenitors *B. oleracea*, 11,930 DEGs were identified in four tissues, including 5233 in stems, 5025 in leaves, 6708 in flowers and 5122 in siliques (Fig. 3). In total, the DEGs between allotetraploid *B. napus* and *B. rapa* were approximately 1.5 times that between *B. napus* and *B. oleracea*; among these, the most different tissue was stems. Specifically, the DEGs in stems between *B. napus* and *B. rapa* were approximately 1.9 times that between *B. napus* and *B. oleracea*. In allotetraploid *B. napus*, more DEGs from both A and C subgenomes were downregulated relative to those in its two diploid progenitors, and an average of 53% (9266 of 17,463) and 52.9% (6312 of 11,930) of DEGs in the A and C subgenomes were downregulated, respectively (Fig. 3).

### Functional classifications of the DEGs

To further explore the gene functional differences between *B. napus* and its two diploid progenitors in four selected tissues, all genes from the A and C genomes were functionally annotated based on eggNOG database. A total of 91% (42,103 of 46,250) and 91.8% (42,017 of 45,758) of the genes from the A and C genomes, respectively, were annotated. Moreover, 54.8% (23,083 of 42,103) and 57.4% (24,102 of 42,017) of the genes from the A and C genomes were annotated to at least one GO term, respectively. Then, the GO functional categories of all DEGs between *B. napus* and its diploid progenitors were investigated using GO functional classification analysis (WEGO). A total of 55 enriched GO terms were identified among DEGs, including three categories: biological process (31 GO terms), molecular function (8 GO terms), and cellular component (16 GO terms) (Figure S1). In the DEGs between *B. napus* and the diploid progenitors *B. rapa*, there were four significant enrichment GO level 2 terms, including growth (GO:0040007), membrane-enclosed lumen (GO:0031974), membrane part (GO:0044425), and organelle part (GO:0044422) (Figure S1). While in the DEGs between *B. napus* and *B. oleracea*, there were seven significant enrichment GO level 2 terms, such as immune system process (GO:0002376), response to stimulus (GO:0050896), extracellular region (GO:0005576), and organelle part (GO:0044422) (Figure S1). In addition, in order to obtain more useful information, the GO enrichment analysis of up−/downregulated DEGs between *B. napus* and its two diploid progenitors was performed separately (Table S1). The upregulated DEGs between *B. napus* and the diploid progenitors *B. rapa* were significantly enriched to the largest number (22) of GO items (Table S1). A majority of upregulated DEGs between *B. napus* and *B. rapa* were identified as biological process GO items, while the other three groups (including downregulated DEGs between *B. napus* and *B. rapa* and up−/down-regulated DEGs between *B. napus* and *B. oleracea*) had a majority of genes identified as cellular component GO items (Table S1). These results showed that the upregulated DEGs might play an important role in biological process (such as developmental process and multicellular organismal process), while the downregulated DEGs might play a critical role in cellular component (such as cell part, organelle part and membrane) in the DEGs between *B. napus* and the diploid progenitors *B. rapa*. However, both upregulated and downregulated DEGs might play a major role in cellular component (such as such as cell part, organelle part and cell junction) in the DEGs between *B. napus* and the diploid progenitors *B. oleracea*.

### KEGG analysis of the DEGs

To identify the metabolic or signal transduction pathways involved in DEGs, all DEGs were annotated to KEGG pathways based on eggNOG database. A total of 12 pathways were significantly enriched (q value ≤0.05) in DEGs between *B. napus* and its diploid progenitors *B. rapa*, such as photosynthesis (ko00195), pentose phosphate pathway (ko00030), and circadian rhythm-plant (ko04712). However, only 4 pathways were significantly enriched in DEGs between *B. napus* and *B. oleracea*, including plant-pathogen interaction (ko04626), photosynthesis (ko00195), photosynthesis-antenna proteins (ko00196), and carbon fixation in photosynthetic organisms (ko00710). Three pathways related to photosynthesis (ko00195, ko00196, ko00710) were enriched in both comparison groups (A_n_A_n_C_n_C_n_ vs A_r_A_r_ and A_n_A_n_C_n_C_n_ vs C_o_C_o_), and these three pathways were downregulated in all four tissues of *B. napus* relative to its diploid progenitors. Furthermore, DEGs between *B. napus* and its diploid progenitors were involved in many plant physiological processes. The specific statistics of KEGG enrichment in every comparison group are shown in Table S2. Moreover, the sum of the TPM values of the differential genes involved in each KEGG pathway was calculated, and the top 5 up- and downregulated pathways are shown in Table S3.

### Homoeolog expression bias in natural allotetraploid *B. napus*

**Fig. 4:**
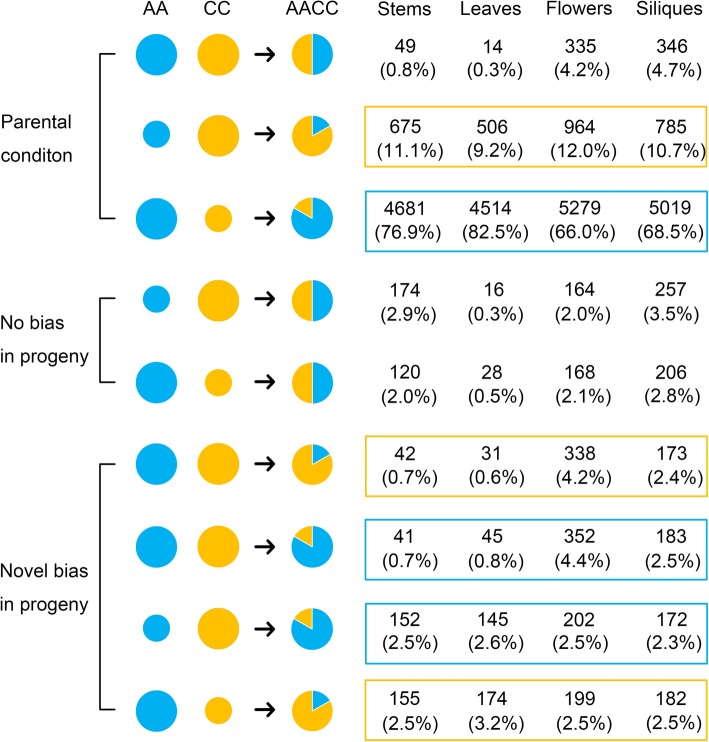
Homoeolog expression bias in the four tissues of the natural allotetraploid *B. napus*. The relative expression levels of the homologous gene pairs were modeled by the size of the circles in the diploid progenitors *B. rapa* (AA) and *B. oleracea* (CC) or the area ratio of the circles in *B. napus* (AACC). The number of homologous gene pairs were listed in this figure. Homologous gene pairs that showed biased expression towards A subgenome in *B. napus* were marked with a blue box, and gene pairs displayed C-biased were marked with a yellow box

Previous studies have shown that the duplicated gene pairs in allotetraploids might display homoeolog expression bias, where bias refers to the preferential and high expression of one homoeolog relative to the other homoeolog [14, 48–50]. To study the homoeolog expression bias in the natural allotetraploid *B. napus*, the expression levels of 27,609 homologous gene pairs from *B. rapa* and *B. oleracea* were analyzed. These homologous gene pairs were obtained using a perl script (Additional file 6). Then, DESeq2 was used to analyze whether there were expression differences between these gene pairs. Homologous gene pairs that met the condition of |log_2_ fold change| ≥ 1 and padj ≤0.001 were considered to be differentially expressed gene pairs. Compared with the diploid progenitors, the homologous gene pairs between the two subgenomes of *B. napus* were divided into three categories, including the parental condition, no bias, and novel bias in progeny (Fig. 4). As shown in Fig. 4, the overwhelming majority of gene pairs (an average of 86.7%) from the two subgenomes of natural allotetraploid *B. napus* maintained their expression pattern in two diploid progenitors, and this feature was most obvious in leaves (92%) and least obvious in flowers (82.2%). Moreover, an average of 4% gene pairs that already had expression bias in the two diploid progenitors reverted to no bias expression in *B. napus*, and only 0.8% homologous gene pairs in leaves of *B. napus* had this reversion (Fig. 4). In addition, an average of 9.2% homologous gene pairs displayed novel bias in *B. napus*, and this phenomenon was most common in flowers (13.6%). According to the statistics on the number of gene pairs with A−/C-bias or no bias expression in allotetraploid, 78.1, 15.4 and 6.5% of the homologous gene pairs showed A-bias, C-bias and no bias expression, respectively (Fig. 4). This result seems to indicate that a highly unbalanced biased expression was observed in the natural allotetraploid *B. napus*, which had a preference toward the A subgenome (A-bias vs C-bias = 78.1% vs 15.4%). However, further analysis showed that this is simply a parental legacy. In detail, the number of gene pairs in two diploid progenitors were also counted, and 78, 15.5 and 6.6% of the orthologous gene pairs showed A > C, A < C and A = C in gene expression, respectively (Fig. 4).

### Expression level dominance (ELD) in the natural allotetraploid *B. napus*

**Fig. 5:**
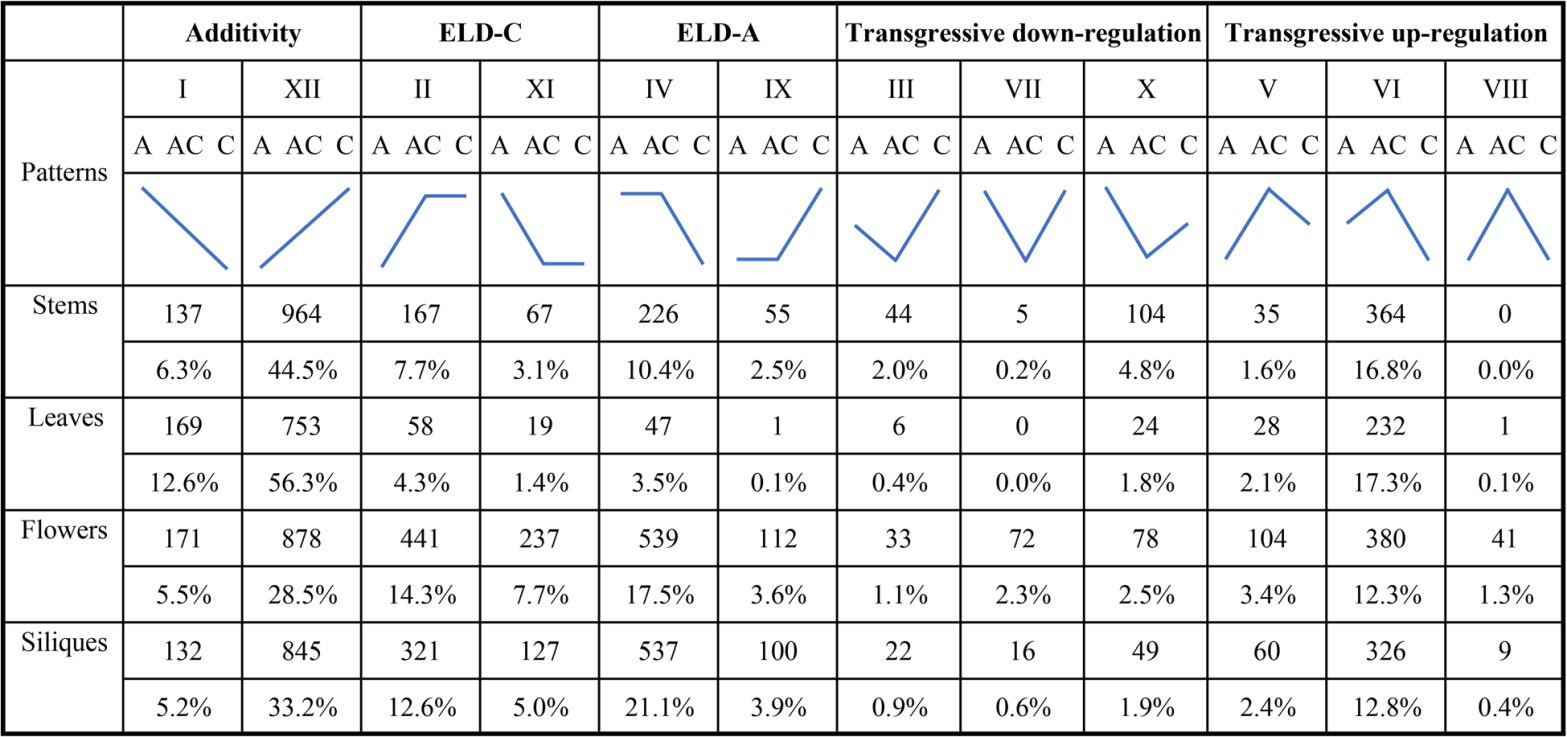
The 12 possible differential expression states in *B. napus* relative to its two diploid progenitors. This classification method refers to Yoo et al. [48]. According to the different expression patterns, genes in *B. napus* and their progenitors were divided into additive and non-additive expression genes, and the latter is further classified into expression level dominance (ELD) and transgressive expression genes

In addition to homoeolog expression bias in gene pairs, ELD has been frequently described in the study of allopolyploidy [14, 48, 50–52]. Homoeolog expression bias mainly focused on the relative expression levels of the individual homologs, whereas ELD primarily focused on the total expression levels of homologous gene pairs in allopolyploids compared to their relative expression levels in its two parents [14, 48, 50–52]. To study additivity, transgressive expression and ELD in the four tissues of the natural allotetraploid *B. napus*, the homologous gene pairs were classified into 12 categories by comparing the total expression levels of the gene pairs in *B. napus* relative to its two diploid progenitors [48]. Overall, an average of 48% homologous gene pairs exhibited additivity expression (categories I and XII), and the remaining 29.7 and 22.3% of gene pairs showed ELD (categories II, XI, IV and IX) and transgressive expression (categories III, VII, X, V, VI and VIII), respectively, in natural allotetraploid *B. napus* (Fig. 5). More A-expression level dominance (ELD-A) homologous gene pairs (categories IV and IX with an average of 15.7%) were observed in *B. napus* than C-expression level dominance (ELD-C) gene pairs (categories II and XI with an average of 14%, Fig. 5). Therefore, the expression of gene pairs in the natural allotetraploid *B. napus* displayed ELD bias toward *B. rapa*. In addition, more gene pairs showed obvious transgressive upregulation expression (categories V, VI and VIII with an average of 17.6%) rather than downregulation (categories III, VII and X with an average of 4.7%, Fig. 5).

Homologous gene pairs that exhibited additivity expression were the most abundant in leaves (68.9%) and the least abundant in flowers (34%). Gene pairs showed more ELD-A than ELD-C in stems and siliques (13% ELD-A vs 10.8% ELD-C in stems and 25% ELD-A vs 17.6% ELD-C in siliques), while gene pairs displayed more ELD-C than ELD-A in leaves and flowers (3.6% ELD-A vs 5.8% ELD-C in leaves and 21.1% ELD-A vs 22% ELD-C in flowers, Fig. 5). Tissues with more ELD-A genes (stems and siliques) were indeed closer to *B. rapa* in morphology (Fig. 1) in *B. napus*, and it is speculated that ELD-A genes were involved in the regulation of plant tissue morphogenesis. The GO enrichment analysis of ELD-A/−C genes in each tissue is shown in Figure S2, and the results indicated that they were mainly enriched in two parts (cellular component and biological process). In addition, the proportion of gene pairs with transgressive upregulation expression was higher than that of gene pairs with downregulation in all four tissues of *B. napus*.

### Relationship between individual homoeolog expression level and ELD

**Fig. 6:**
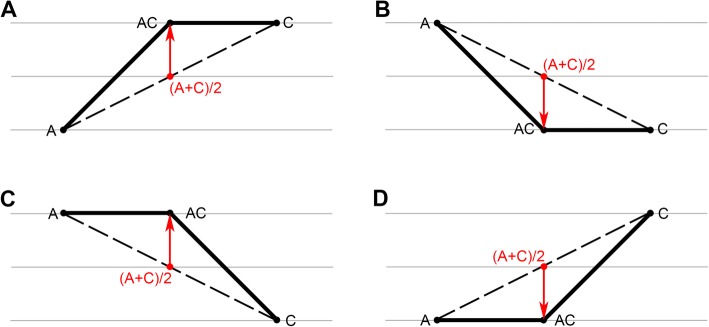
The explanations for ELD-A or ELD-C. If the allotetraploid *B. napus* (AC) maintains the progenitor’s expression pattern, its expression pattern should be additive (red text). The expression level of AC was higher than (A + C)/2, which could be explained by the upregulation of at least one homolog from progenitor A or C (**a** & **c**). Moreover, the expression level of AC was lower than (A + C)/2, which could be explained by the downregulation of at least one homolog from progenitor A or C (**b** & **d**)

Individual homoeolog expression levels in four tissues of *B. napus* relative to diploid progenitors were investigated to explain the ELD phenomenon. More modifications were observed in the A homoeolog (88, 16, 293, and 169 genes in stems, leaves, flowers, and siliques, respectively) than the C homoeolog (58, 13, 185, and 125 genes) (Table 3). The dominant progenitor has a higher expression level than the nondominant progenitor in categories II and IV (Fig. 5), and these findings could be explained by the upregulation of at least one homolog from dominant or nondominant progenitor (Fig. 6a and c, Table 3). In contrast, the dominant progenitor has a lower expression level than the nondominant progenitor in categories XI and IX (Fig. 5), and these findings could be explained by the downregulation of at least one homolog from dominant or nondominant progenitor (Fig. 6b and d, Table 3). Statistics showed that the up−/downregulation of homologs from nondominant progenitors always exceeded the homologs from dominant progenitors, except for category IX in flowers (Table 3).

**Table 3:** Homoeolog expression levels of genes that were displayed ELD in four tissues of *B. napus*

Homoeolog regulation in ***B. napus***	Stems				Leaves				Flowers				Siliques			
	II	XI	IV	IX	II	XI	IV	IX	II	XI	IV	IX	II	XI	IV	IX
Both homoeologs up-regulated	3	0	2	0	2	0	0	0	5	0	8	0	4	0	5	1
Only A homoeolog up-regulated	28	0	0	0	1	0	0	0	63	0	9	3	44	0	3	1
Only C homoeolog up-regulated	0	0	1	0	0	0	1	0	0	0	20	1	1	0	7	1
A up- and C down-regulated	11	0	0	0	3	0	0	0	20	0	7	1	22	0	1	0
Both homoeologs down-regulated	2	17	0	9	1	3	0	0	8	41	0	14	7	17	0	13
Only A homoeolog down-regulated	1	11	0	2	1	2	0	0	18	64	0	16	6	25	0	11
Only C homoeolog down-regulated	3	3	0	5	0	0	0	0	10	10	12	12	6	8	6	17
A down- and C up-regulated	0	0	2	0	0	0	3	0	5	4	6	1	2	1	6	0
Homoeolog from non-dominant progenitor up-regulated	42	–	5	–	6	–	4	–	88	–	34	–	70	–	18	–
Homoeolog from dominant progenitor up-regulated	3	–	2	–	2	–	0	–	10	–	24	–	7	–	9	–
Homoeolog from non-dominant progenitor down-regulated	–	28	–	14	–	5	–	0	–	109	–	27	–	43	–	30
Homoeolog from dominant progenitor down-regulated	–	20	–	11	–	3	–	0	–	51	–	31	–	25	–	24

## Discussion

Polyploidization is highly common in the evolutionary history of plants, especially angiosperms [2, 53]. How naturally occurring allopolyploids coordinate more than one set of diverged genomes and regulate their interactions after undergoing hybridization and polyploidization is an intriguing question. Allotetraploid *B. napus* was formed by the natural hybridization and polyploidization of two diploid progenitors (*B. rapa* and *B. oleracea*) approximately 7500 years ago [21], and these three species serve as a typical system for exploring the effects of hybridization and genome duplication on allopolyploid genomes [37]. After approximately 7500 years of evolution, the two immediate ancestral parents of natural *B. napus* were already unavailable, thus we had to select two sequenced species with the same genome type as the two ancestral parents for this study. The disadvantage of this choice is that the two species also evolved within the diploid during the approximately 7500 years, and the most authentic gene expression patterns in the ancestral parents of natural *B. napus* cannot be accurately described. However, previous studies have shown that *B. rapa* and *B. oleracea* formed around 4.6 million years ago [46], and 7500 years of evolutionary history is relatively short for them compared to 4.6 million years. Therefore, the set of materials we selected can still be used to explore the changes in the expression patterns of homologous gene pairs in allotetraploid natural *B. napus* after integrating the two sets of genomes. In addition, different human artificial selection on these three species can lead to differences in the evolution of their genomes, which is an unavoidable limitation of working with these three species. Since the formation of *B. napus*, gene loss and gene expression differentiation may have occurred [21]. This study analyzed the RNA-seq data from four major tissues (stems, leaves, flowers and siliques) of naturally occurring *B. napus* and its two diploid progenitors to investigate the effects of natural hybridization and polyploidization on gene expression in *B. napus*. In particular, this study also investigated the ELD and homoeolog expression bias of natural allotetraploid *B. napus*.

### Transcriptomic shock during allopolyploidization in *B. napus* was relieved

Given that *B. napus* has undergone genome-wide duplication, it is a significant challenge to identify the expression patterns of *B. napus* homologous genes using early research techniques, such as Arabidopsis-specific microarrays and short single-end sequencing [20, 30, 32, 33]. In this study, 2 × 150 bp paired-end sequencing was used, which was recognized to be more capable in the identification of homologous genes and the study of the whole transcriptome [20]. Most (on average, approximately 71%) clean reads could be uniquely mapped to unique regions of A or C genomes/subgenomes (Table 1). Only the uniquely mapped reads were further analyzed in this study. Moreover, in previous studies of Brassica polyploids and their parents or progenitors, only the genome of one species (usually *B. rapa*) was used as the reference genome for all species [20, 54], making it difficult to distinguish A and C homologous genes in allotetraploid *B. napus*. In this study, *B. rapa* (A genome), *B. oleracea* (C genome) and integrated genome (A-C genome) sequences were used as the reference genomes of three species; therefore, the expression patterns of homologous genes can be accurately determined. Although the mapping rate of A and C reference genomes is different, it only affects the number of detected genes, not the gene expression levels of detected genes, so it does not have a huge impact on the analysis of gene expression patterns of homologous gene pairs. Previous studies have shown that the gene expression pattern of resynthesized *B. napus* changed widely in the early stage of its formation (approximately one-third of the expressed genes were DEGs in resynthesized *B. napus* and its two diploid parents); in other words, ‘transcriptomic shock’ occurred in resynthesized allotetraploid *B. napus* [20]. The phenomenon of ‘transcriptomic shock’ [55, 56] is common in allopolyploids, such as cotton [48], wheat [14] and *Senecio* [55]. In this study, only approximately one-fifth of the expressed genes were DEGs in natural allotetraploid *B. napus* and its two diploid progenitors (Table 2 and Fig. 3), which indicated that the numbers of DEGs in natural *B. napus* were decreased and the ‘transcriptomic shock’ phenomenon might be alleviated during the long evolutionary process.

The increase of gene and genome dosages in allopolyploidy often leads to some problems, such as genome instabilities and chromosome imbalances. Thus, allopolyploids must establish a compatible relationship between two different genomes, resulting in a series of changes, such as changes in genome structure and reprogramming of homologous gene expression [12]. According to a previous study [20], up to 85% of DEGs between the resynthesized *B. napus* and its diploid parent were downregulated, while in our study, the number of upregulated and downregulated DEGs between the natural *B. napus* and its diploid progenitors was almost the same. While the number of DEGs decreased, the number of upregulated and downregulated genes also tended to balance. The reprogramming phenomenon of gene expression in the allopolyploid *B. napus* is gradually balanced and stable in the long evolutionary process.

### Overwhelming gene pairs showed expression bias with an obvious preference toward the a subgenome in naturally cultivated *B. napus*

Previous studies have shown that some duplicate genes may have different expression patterns as a result of genome replication [17, 48, 51]. In this study, homoeolog expression bias and ELD were used to describe the changes in the expression patterns of duplicate genes in the naturally cultivated allopolyploid *B. napus*. Homologous expression bias refers to the unequal contribution of A and C homoeologs to total gene expression in natural allopolyploid *B. napus* (the preferential expression of one homoeolog relative to the other) [20]. Moreover, homologous expression bias has been reported in many allopolyploids, such as wheat [14, 57, 58] and cotton [48, 59, 60]. A previous study documented that approximately 36.5% of the homologous gene pairs exhibited expression bias in resynthesized *B. napus* [20], while in this study, an average of 93.5% of the homologous gene pairs showed expression bias in four tissues of the natural allotetraploid *B. napus* (Fig. 4). Similar studies have also been carried out in allotetraploid cotton [59], and the results have shown that genome expression biases in natural allotetraploid (70.1%) were more than in resynthesized cotton (30.5%). The difference in expression bias between natural and resynthesized *B. napus* may be caused by the long-term domestication process affecting the expression patterns of duplicated genes.

In addition, an obvious unbalanced biased expression was found in natural *B. napus*, with 78.1% of the gene expression biased toward the A subgenome and 15.4% biased toward the C subgenome (Fig. 4), even if it’s just a parental legacy. Previous studies have shown that there was a hierarchy of nucleolar dominance (subgenome B > A > C) in three *Brassica* allotetraploids. Specifically, *B. juncea* and *B. carinata* expressed rRNA genes from their B subgenome and *B. napus* expressed from their A subgenome [61–63]. Furthermore, different subgenome stability (subgenome B > A > C) were observed in synthesized *Brassica* allohexaploids (2n = 54, AABBCC) [64]. Therefore, both nucleolar dominance and subgenome stability showed that the A subgenome had more advantages than the C subgenome. These conclusions supported our results that homoeolog expression bias of gene pairs in natural *B. napus* showed a preference toward the A subgenome.

### Gene expression showed different ELD biases in different tissues in natural *B. napus*

In this study, ELD refers to the fact that the total expression level of a homologous pair is statistically identical to that of only one of the two diploid progenitors in natural *B. napus*. The term ‘ELD’ was first proposed by Grover et al. [50] and was previously referred to as ‘genome advantage’ [51]. The expressed homoeolog pairs were classified into 12 categories according to the method reported by Yoo et al. [48]. Overall, an average of 29.7% of the expressed homoeolog pairs exhibited ELD in natural *B. napus* (Fig. 5). An interesting phenomenon is that the ELD bias varies from tissue to tissue. More gene pairs in stems and siliques showed ELD-A, whereas the opposite was true in leaves and flowers (Fig. 5). A previous study found that there were more ELD-A than ELD-C in the leaves of resynthesized *B. napus* [20]. There are two possible reasons for this phenomenon. One reason is that the leaves to be analyzed from plants growing at different times (young leaves of 6-month-old plants were analyzed in our study, while the fourth true leaves of 40-day-old materials were sent in Wu et al.’s study). The other is that the expression of genes in natural *B. napus* has changed over the long course of domestication compared to that of genes in resynthesized *B. napus*. Moreover, the relationship between individual homoeolog expression levels and ELD was investigated. When the dominant progenitor has a higher expression level than the nondominant progenitor, the ELD could be explained by the upregulation of at least one homolog from dominant/nondominant progenitor (Fig. 6a and c, Table 3). In contrast, when the dominant progenitor has a lower expression level, the ELD could be explained by their downregulation (Fig. 6b and d, Table 3).

In addition, it is worth noting that more transgressive upregulation expression, rather than downregulation expression, was observed in gene pairs of natural *B. napus*. In this study, transgressive expression refers to the fact that the total expression level of homologous gene pairs in natural *B. napus* is statistically higher or lower than that of gene pairs in two diploid progenitors. The current study indicated that an average of 22.3% of the gene pairs showed transgressive expression. Among these pairs, more gene pairs showed significant upregulation (17.6% on average), rather than downregulation (4.7% on average, Fig. 4). Previous studies on resynthesized *B. napus* showed that 9% of the gene pairs were transgressive expression, and almost all of them were upregulated (8.7% transgressive upregulation vs 0.3% transgressive downregulation) [20]. Obviously, 79% (17.6% of the 22.3%) of the transgressive expression gene pairs were upregulated in natural *B. napus*, but 96% (8.7% of the 9%) were upregulated in resynthesized *B. napus*. These results suggested that compared with the phenomenon in resynthesized *B. napus*, the long process of domestication may not change the advantage of transgressive upregulation of the expression of gene pairs in natural *B. napus* but may weaken it instead.

What’s more, the KEGG analysis of DEGs showed that three pathways related to photosynthesis (ko00195, ko00196, ko00710) were downregulated in all four tissues of *B. napus* relative to its diploid progenitors, suggesting that the photosynthesis of natural *B. napus* might not be as active as that of its two diploid progenitors, which still needs to be verified by follow-up experiments. Furthermore, DEGs involved in many plant physiological processes, which were mentioned in KEGG analysis, may also be involved in plant morphogenesis, which may provide evidence for the morphological differences in the stems, leaves, flowers and siliques of *B. napus* compared with its diploid progenitors (Fig. 1).

## Conclusions

In this study, the global gene pair expression patterns in four major tissues (stems, leaves, flowers and siliques) of natural allotetraploid *Brassica napus* (A_n_A_n_C_n_C_n_) and its two diploid progenitors, *B. rapa* (A_r_A_r_) and *B. oleracea* (C_o_C_o_), were analyzed using an RNA sequencing approach. The results showed that the ‘transcriptomic shock’ phenomenon was alleviated in natural *B. napus* after approximately 7500 years of natural domestication. In addition, the results of homoeolog expression bias analysis in natural *B. napus* indicated that 86.7% of the orthologous gene pairs in *B. napus* maintained their expression pattern in two diploid progenitors, and most gene pairs showed expression bias with a preference toward the A subgenome. Moreover, the results of expression level dominance (ELD) analysis in natural *B. napus* showed that the ELD bias varies from tissue to tissue, specifically, more gene pairs in stems and siliques showed ELD-A, whereas the opposite was true in leaves and flowers. Taken together, these results may provide a comprehensive understanding of the changes in homologous gene expression patterns in natural *B. napus* after approximately 7500 years of evolution and domestication and may help to characterize allopolyploidy.

## Methods

### Plant materials

Three plant materials, including the natural allotetraploid *B. napus* (cv. Darmor, catalogue number: 00003410, 2n = 4x = 38, A_n_A_n_C_n_C_n_) and its two diploid progenitors, *B. rapa* (cv. Chiifu, catalogue number: 00008965, 2n = 20, A_r_A_r_) and *B. oleracea* (cv. Jinzaosheng, catalogue number: V04A0086, 2n = 18, C_o_C_o_), were obtained from the Oil Crops Research Institute, Chinese Academy of Agricultural Sciences, China. The plants were planted randomly under natural conditions (outside) in the greenhouse at Wuhan University, China. Soil collected from local environments and mixed with compound fertilizer, which was watered manually to keep the soil moist. The main components of the compound fertilizer are N (17%), P (5%) and K (7%). The amount of compound fertilizer in each pot (diameter: 40 cm) is about 8 g. Artificial pollination was performed on each plant material. To prevent the contamination from exogenous pollen, some inflorescences were bagged before blossom. Four tissues of 6-month-old plants, including inflorescence stems, young leaves, blooming flowers and siliques (10 DAP, days after pollination), were collected at the same time (approximately 10 am) and frozen in liquid nitrogen quickly for RNA extraction. Three biological replicates were performed.

### RNA extraction, cDNA library construction and transcriptome sequencing

Total RNA was extracted using TRIzol reagent (Invitrogen, USA) according to the manufacturer’s protocol and then treated with RNase-free DNase I (Thermo Scientific, USA) to remove the residual DNA. An Agilent 2100 Bioanalyzer (Agilent RNA 6000 Nano Kit) was used to assess the yield and purity of the total RNAs. Criteria to determine whether extracted RNA can be used for future RNA-seq library building, including the content of total RNAs ≥1 μg, the concentration of total RNAs ranged from 40 to 2500 ng/μl, the RIN value ≥6.5, the OD_260/280_ value ≥1.8, the OD_260/230_ value ≥1.8 and the 28S/18S value ≥1.0. A total of 36 RNA samples were used to construct the RNA-seq libraries. The RNA sequencing library was constructed according to the eukaryotic transcriptome library construction protocol provided by BGI (SOP-SS-038, A0). Specifically, 1 μg of total RNA was collected, and the mRNAs containing polyA tails were enriched with oligo-dT magnetic beads to construct the library. The length of the inserted fragment ranged from 250 to 350 bp. The cDNA library was constructed separately for each sample. The libraries were then sequenced using the Illumina HiSeq™X-Ten platform. Two indicators (Q20 and Q30), which indicated the percentage of bases having a mass value of not less than 20 or 30, were used to represent sequencing accuracy. To obtain clean reads, the adapter sequences and the low-quality sequences were filtered out from row reads using software SOAPnuke (v1.4.0) [65] and Trimmomatic (v0.36) [66]. All clean reads of this study were deposited in the NCBI database (accession number: SRR7816633-SRR7816668).

### Alignment of clean reads to reference genomes and normalized expression levels

All clean reads were aligned to the reference genomes using software HISAT2 (v2.1.0, default parameters) [67]. *B. rapa* (cv. Chiifu-401-42, A_r_A_r_) genome v3.0 (http://brassicadb.org/brad/datasets/pub/BrassicaceaeGenome/Brassica_rapa/V3.0/Brapa_sequence_v3.0.fasta.gz) and *B. oleracea* (var. *capitata*-02-12, C_o_C_o_) genome v1.1 (http://brassicadb.org/brad/datasets/pub/BrassicaceaeGenome/Brassica_oleracea/Bol_Chromosome_V1.1/BOL.seq.lst.new.chr20110802_check.fa.gz) were used as the reference genomes for Chiifu and Jinzaosheng, respectively. Moreover, the A_r_A_r_ and C_o_C_o_ genomes described above were integrated and used as the reference genome for the natural allotetraploid Darmor. The above methods were determined based on the following considerations: 1) the study [21] showed that the A_n_ and C_n_ sub-genomes of *B. napus* were highly homologous with the A_r_ genome of *B. rapa* and the C_o_ genome of *B. oleracea* respectively, and it is feasible to map the RNA-seq reads of *B. napus* to the genomes of *B. rapa* and *B. oleracea*; 2) if reads were mapped to their corresponding reference genomes, four groups of homologous gene pairs (Bra*-Bol*, BnA*-BnC*, BnA*-Bra*, BnC*-Bol*) need to be identified for data analysis, as the number of pairing increases, many homologous gene pairs are lost, so covering too few homologous gene pairs may lead to a one-sided conclusion; 3) the study of Wu et al. [20] provides us with a good reference, that is, only a set of homologous gene pairs, namely Bra*-Bol*, can be found in three species to simplify the scientific problem and avoid the disadvantages mentioned above. Only uniquely mapped reads were considered when analyzing the data. The software featureCounts (v1.6.1) [68] was used to quantify the expression of genes by quantifying the number of reads mapped to reference genomes. The expression level of a gene was normalized by the TPM (transcripts per million reads).

### Analysis of differentially expressed genes (DEGs) and gene annotation

The DESeq2 (version: DESeq2_1.20.0) method [69] was used to identify DEGs, and genes with |log_2_ fold change| ≥ 1 and padj (adjusted *P* value) ≤ 0.001 were defined as DEGs in this study. All genes were annotated using eggNOG-mapper [70] based on eggNOG 4.5 orthology data [71] in the eggNOG database (http://eggnog5.embl.de/). Online website WEGO 2.0 (http://wego.genomics.org.cn) [72] was performed for GO (gene ontology) functional classification.

### Analysis of homoeolog expression bias and ELD

To study the features of homoeolog expression bias and ELD in the natural allotetraploid *B. napus*, the expression levels of 27,609 *B. rapa*-*B. oleracea* orthologous gene pairs identified by a perl script (Additional file 6) were monitored. There are three steps to identify the orthologous gene pairs between *B. rapa* and *B. oleracea*: 1) All protein sequences of *B. oleracea* (http://brassicadb.org/brad/datasets/pub/BrassicaceaeGenome/Brassica_oleracea/Bol_Chromosome_V1.1/Scaffold.seq.110729_check.pep.gz) were used as query sequences to perform Blastp based on the *B. rapa* protein database (http://brassicadb.org/brad/datasets/pub/BrassicaceaeGenome/Brassica_rapa/V3.0/Brapa_genome_v3.0_pep.fasta.gz); 2) conversely, using all protein sequences of *B. rapa* (http://brassicadb.org/brad/datasets/pub/BrassicaceaeGenome/Brassica_rapa/V3.0/Brapa_genome_v3.0_pep.fasta.gz) as query sequences to do another Blastp based on the *B. oleracea* protein database (http://brassicadb.org/brad/datasets/pub/BrassicaceaeGenome/Brassica_oleracea/Bol_Chromosome_V1.1/Scaffold.seq.110729_check.pep.gz); 3) a perl script (Additional file 6) was used to extract the orthologous gene pairs. Specifically, a pair of genes was considered an orthologous gene pair only if it had the highest blast score in both blast results. When analyzing the homoeolog expression bias, the expression level of gene pairs in two diploid progenitors (Ar vs Co) and natural allotetraploid *B. napus* (An vs Cn) was compared using DESeq2 (|log_2_ fold change| ≥ 1; padj ≤0.001). The two sets of results were then further compared with each other, and the number of genes in each pattern was counted (Fig. 4). When analyzing the ELD, the sum of the expression levels of gene pairs in natural allotetraploid *B. napus* was compared with their expression levels in two diploid progenitors (An + Cn vs Ar; An + Cn vs Co) using DESeq2 (|log_2_ fold change| ≥ 1; padj ≤0.001). Furthermore, 12 possible patterns of gene expression, including additivity, ELD and transgressive, were summarized according to a previous study [48].

## Supplementary information

**Additional file 1 **: **Figure S1**. Gene ontology (GO) classification of DEGs. **A** GO classification of DEGs between *B. napus* and its diploid progenitors *B. rapa* in four tissues (stems, leaves, flowers and siliques). **B** GO classification of DEGs between *B. napus* and its diploid progenitors *B. oleracea* in four selected tissues. The asterisk (*) represents a statistically significant difference with *p*-value ≤0.05. BP, biological process; MF, molecular function; CC, cell component.

**Additional file 2 **: **Figure S2**. GO enrichment analysis of ELD genes in four tissues of *B. napus.*

**Additional file 3 **: **Table S1**. Gene ontology (GO) classification of up−/down-regulated DEGs.

**Additional file 4 **: **Table S2**. Statistics of the KEGG enrichment in different comparison groups.

**Additional file 5 **: **Table S3**. The top 5 of up−/down-regulated KEGG pathways in *B. napus* and its two diploid progenitors in four tissues.

**Additional file 6 **: The perl script to identify orthologous gene pairs.

## Data Availability

The data supporting the conclusions of our study are included in the manuscript and the additional files. The sequence data generated in the current study have been uploaded to the National Center for Biotechnology Information (NCBI)’s Sequence Read Archive (SRA) repository (https://trace.ncbi.nlm.nih.gov/Traces/sra/sra.cgi?view=announcement) under the accession numbers of SRR7816633-SRR7816668. The web link of the reference genome of *B. rapa* (A_r_A_r_) was http://brassicadb.org/brad/datasets/pub/BrassicaceaeGenome/Brassica_rapa/V3.0/Brapa_sequence_v3.0.fasta.gz. The web link of the reference genome of *B. oleracea* (C_o_C_o_) was http://brassicadb.org/brad/datasets/pub/BrassicaceaeGenome/Brassica_oleracea/Bol_Chromosome_V1.1/BOL.seq.lst.new.chr20110802_check.fa.gz. The web link of eggNOG database was http://eggnog5.embl.de/. The *B. rapa* protein database was constructed from all protein sequences of *B. rapa* (http://brassicadb.org/brad/datasets/pub/BrassicaceaeGenome/Brassica_rapa/V3.0/Brapa_genome_v3.0_pep.fasta.gz). The *B. oleracea* protein database was constructed from all protein sequences of *B. oleracea* (http://brassicadb.org/brad/datasets/pub/BrassicaceaeGenome/Brassica_oleracea/Bol_Chromosome_V1.1/Scaffold.seq.110729_check.pep.gz).

## Abbreviations

DAP: Days after pollination
DEGs: Differentially expressed genes
ELD: Expression level dominance
GO: Gene ontology
M: Million
padj: Adjusted *P* value
R: Pearson correlation coefficient
TPM: Transcripts per million reads
