# Supplementary figures and images for: Homoeolog expression bias and expression level dominance (ELD) in four tissues of natural allotetraploid Brassica napus
Source: BMC Genomics. 2020 Apr 29;21:330. doi: 10.1186/s12864-020-6747-1 (PMC7191788; doi:10.1186/s12864-020-6747-1)

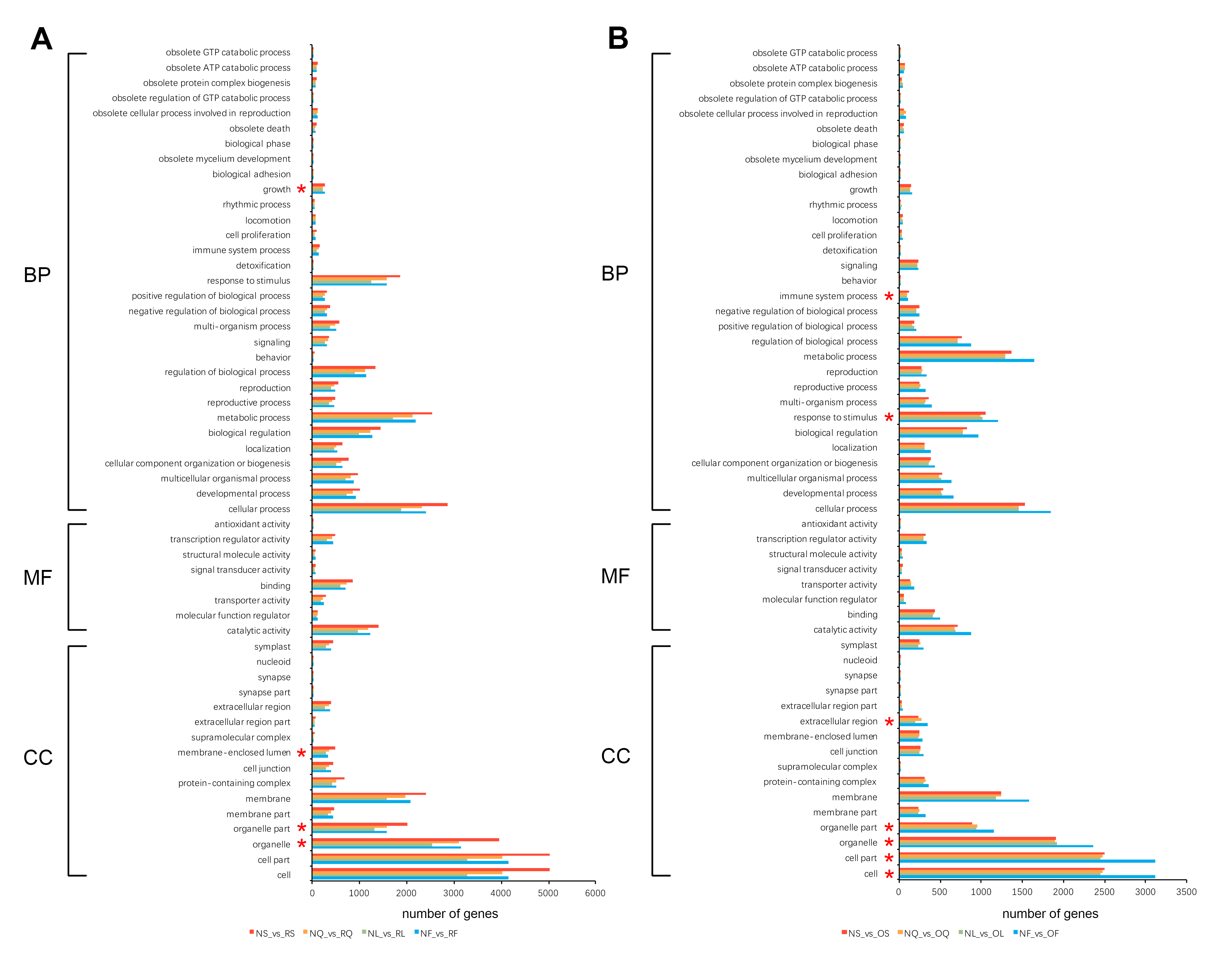

Supplement: Supplementary file 1 — Additional file 1 : Figure S1. Gene ontology (GO) classification of DEGs. A GO classification of DEGs between B. napus and its diploid progenitors B. rapa in four tissues (stems, leaves, flowers and siliques). B GO classification of DEGs between B. napus and its diploid progenitors B. oleracea in four selected tissues. The asterisk (*) represents a statistically significant difference with p-value ≤0.05. BP, biological process; MF, molecular function; CC, cell component. [file 12864_2020_6747_MOESM1_ESM.tif]
