# Supplementary material for: Homoeolog expression bias and expression level dominance (ELD) in four tissues of natural allotetraploid Brassica napus
Source: BMC Genomics. 2020 Apr 29;21:330. doi: 10.1186/s12864-020-6747-1 (PMC7191788; doi:10.1186/s12864-020-6747-1)

**Figure S2-A** ﻿GO enrichment analysis of ELD-A genes in flowers of *B. napus.*


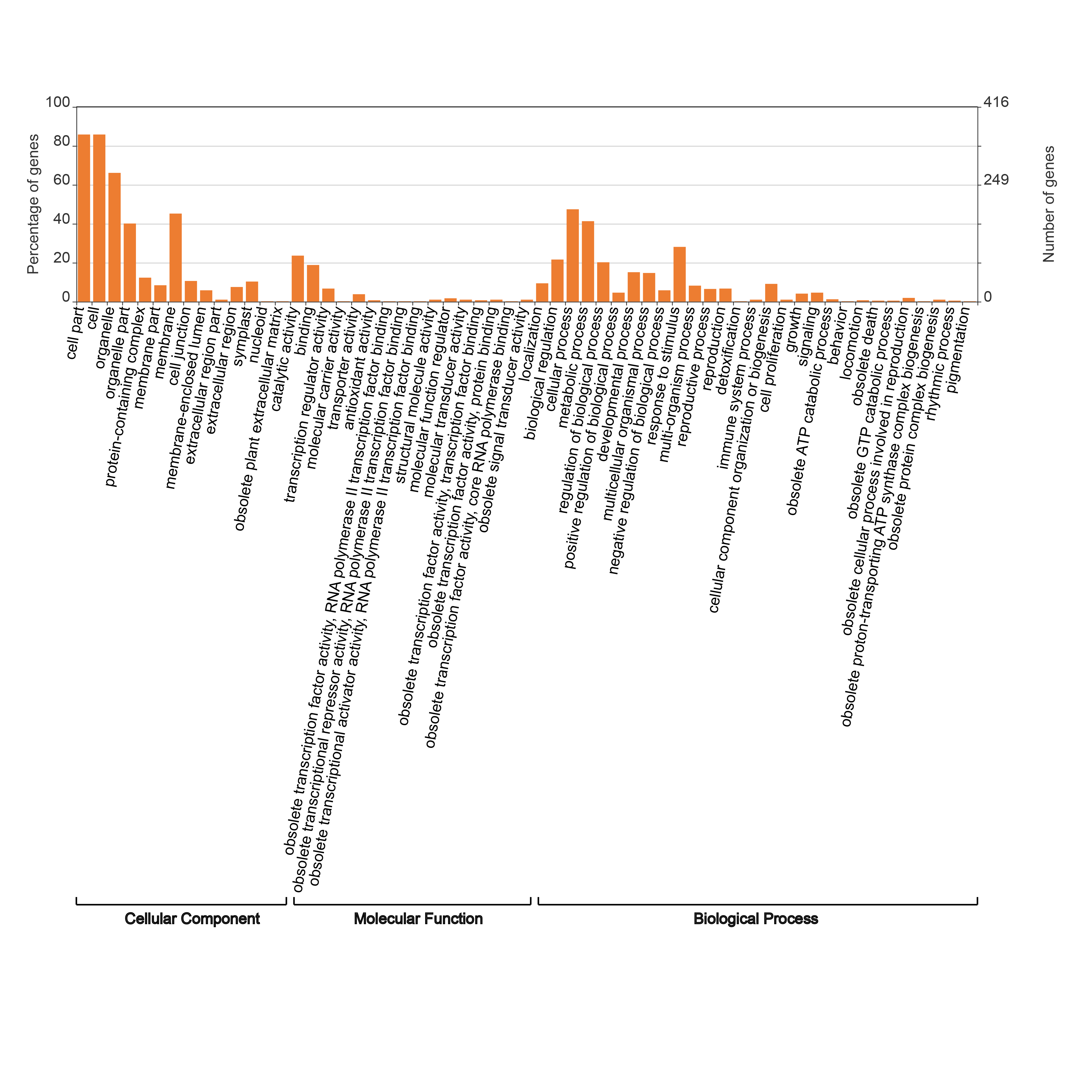


**Figure S2-B** ﻿GO enrichment analysis of ELD-C genes in flowers of *B. napus.*


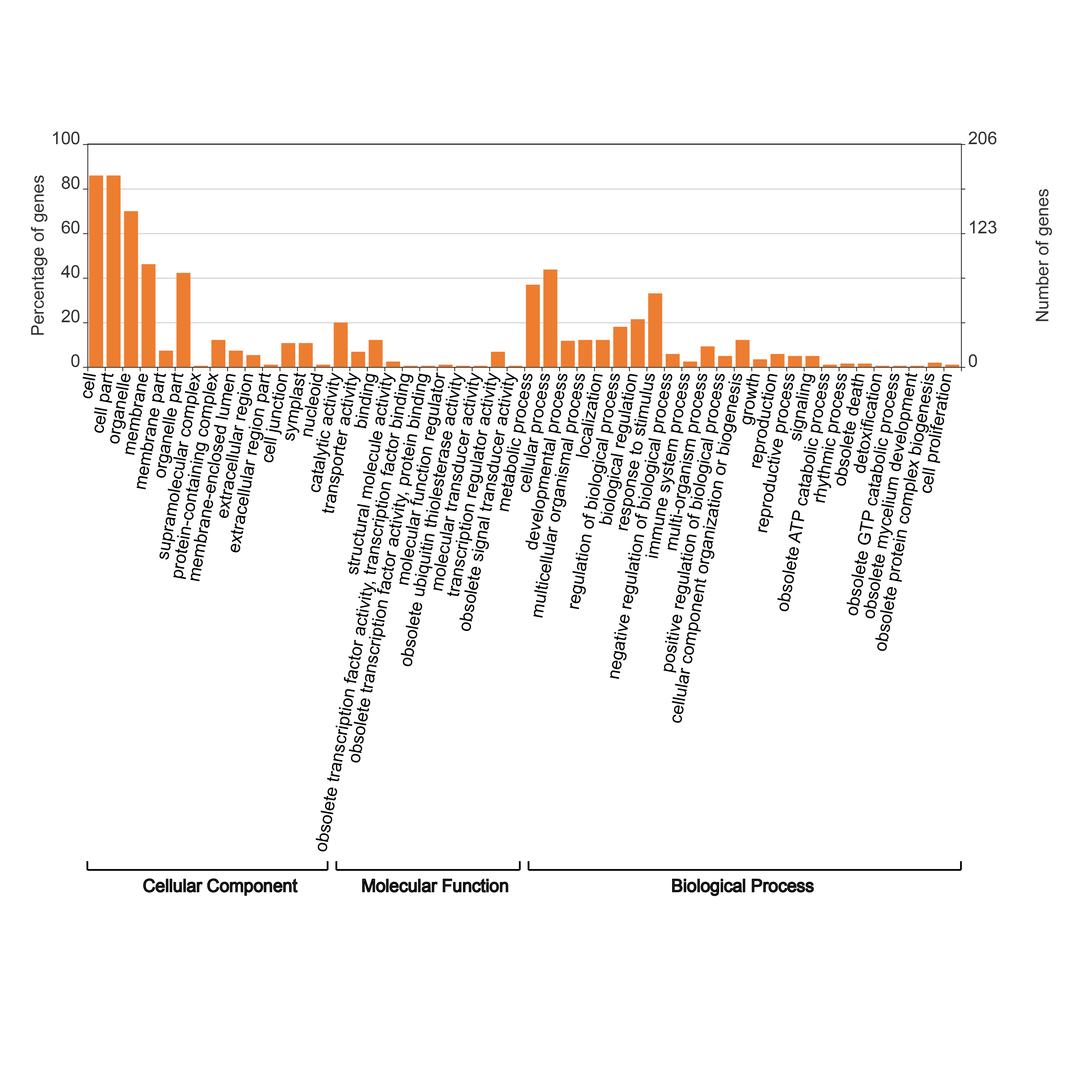


**Figure S2-C** ﻿GO enrichment analysis of ELD-A genes in leaves of *B. napus.*


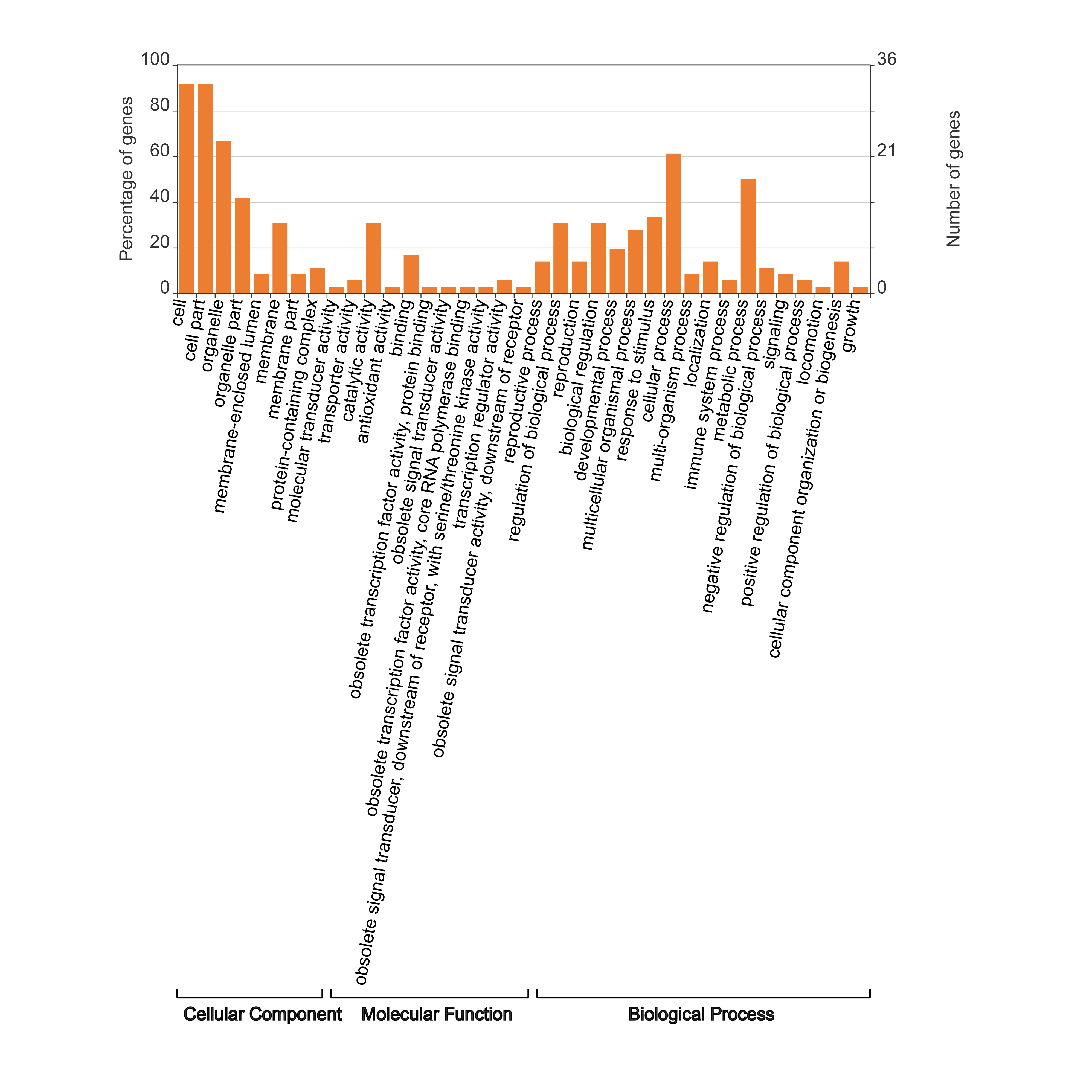


**Figure S2-D** ﻿GO enrichment analysis of ELD-C genes in leaves of *B. napus.*


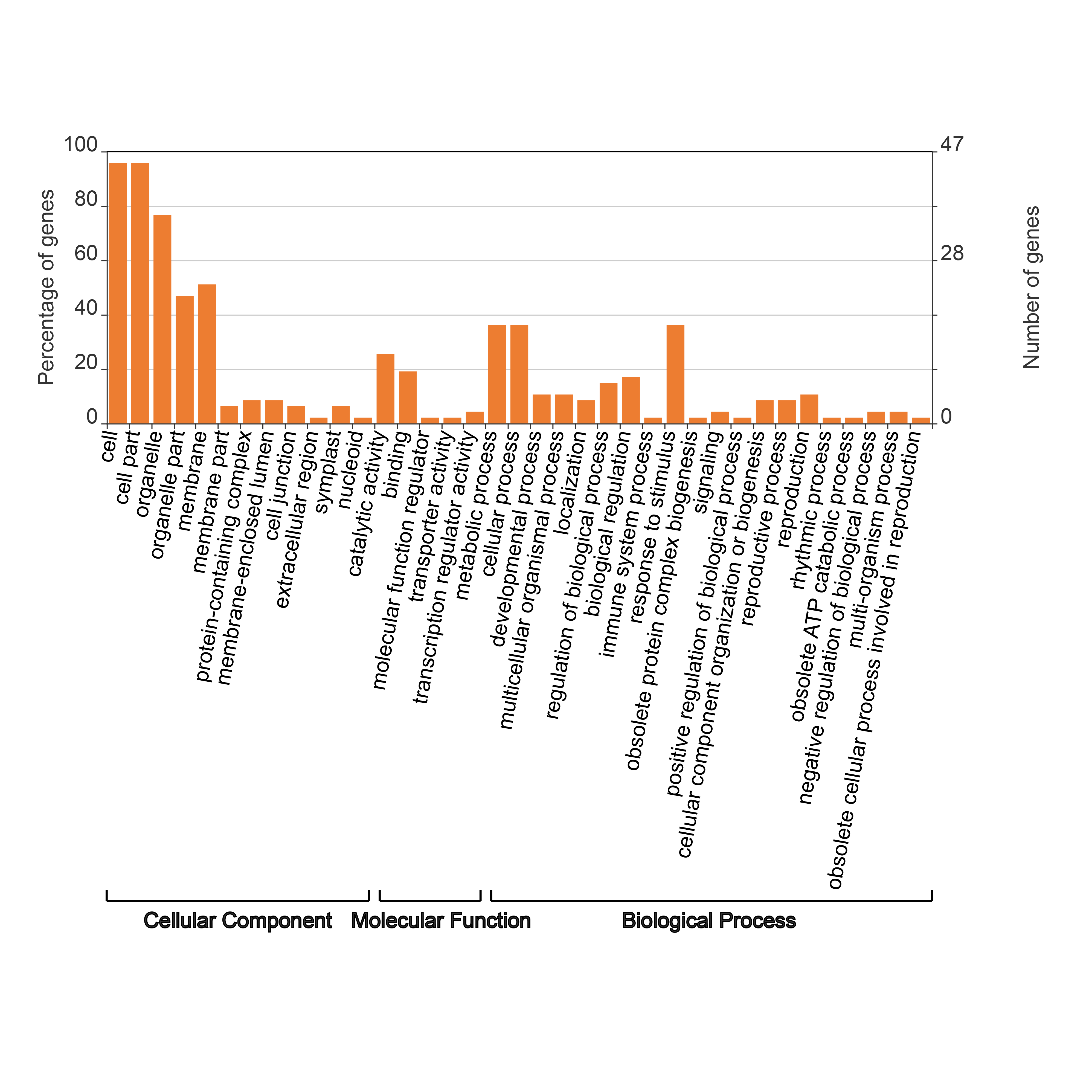


**Figure S2-E** ﻿GO enrichment analysis of ELD-A genes in siliques of *B. napus.*


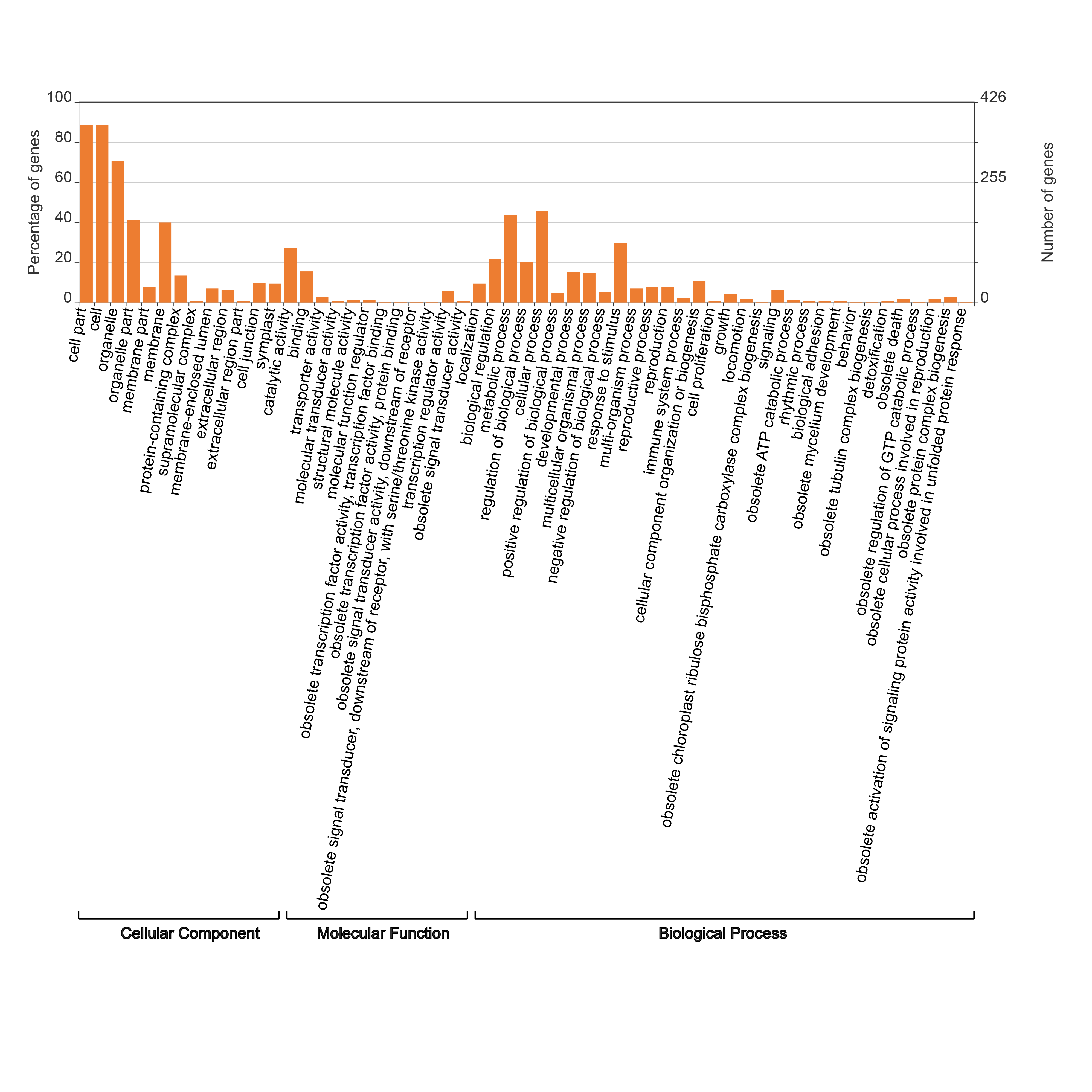


**Figure S2-F** ﻿GO enrichment analysis of ELD-C genes in siliques of *B. napus.*


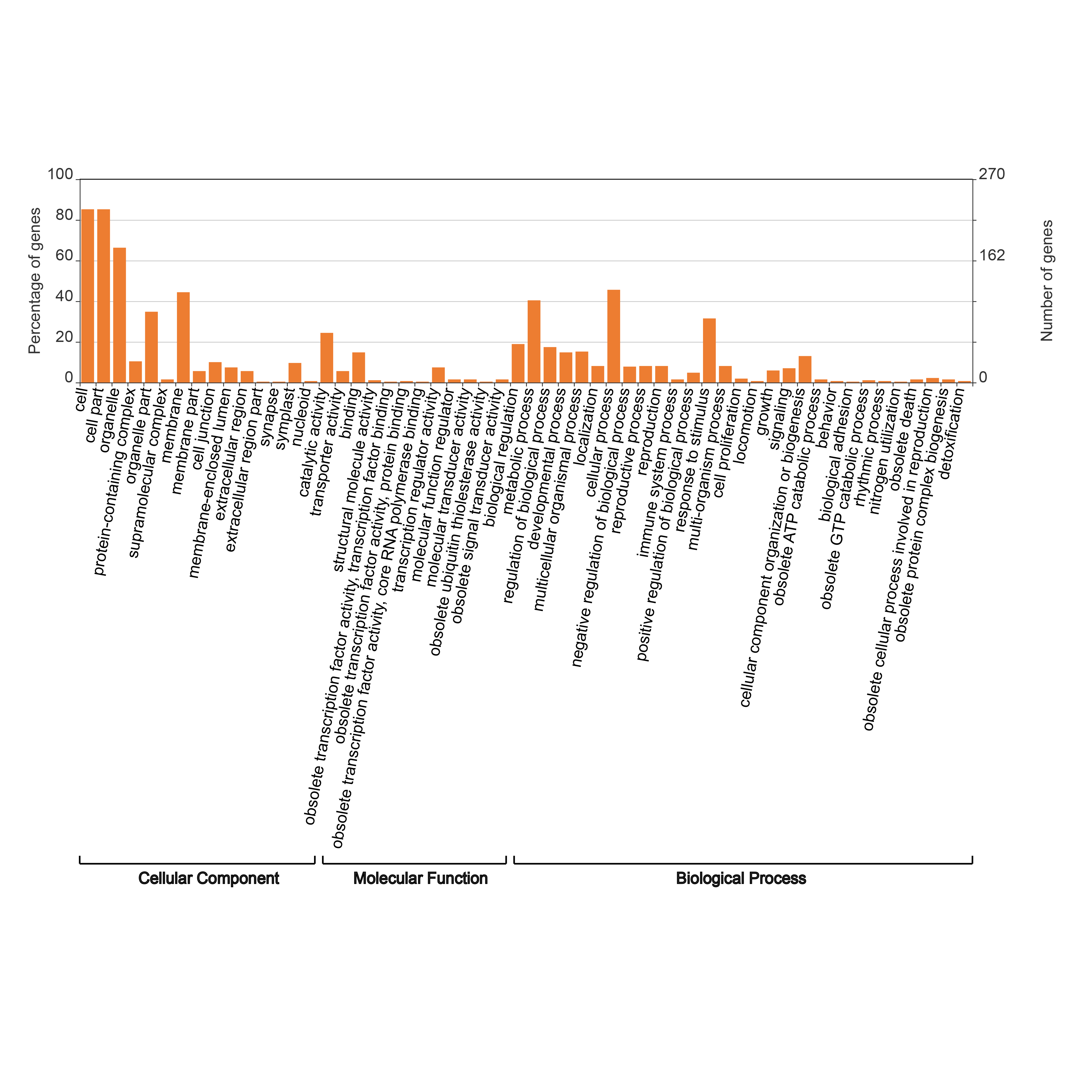


**Figure S2-G** ﻿GO enrichment analysis of ELD-A genes in stems of *B. napus.*


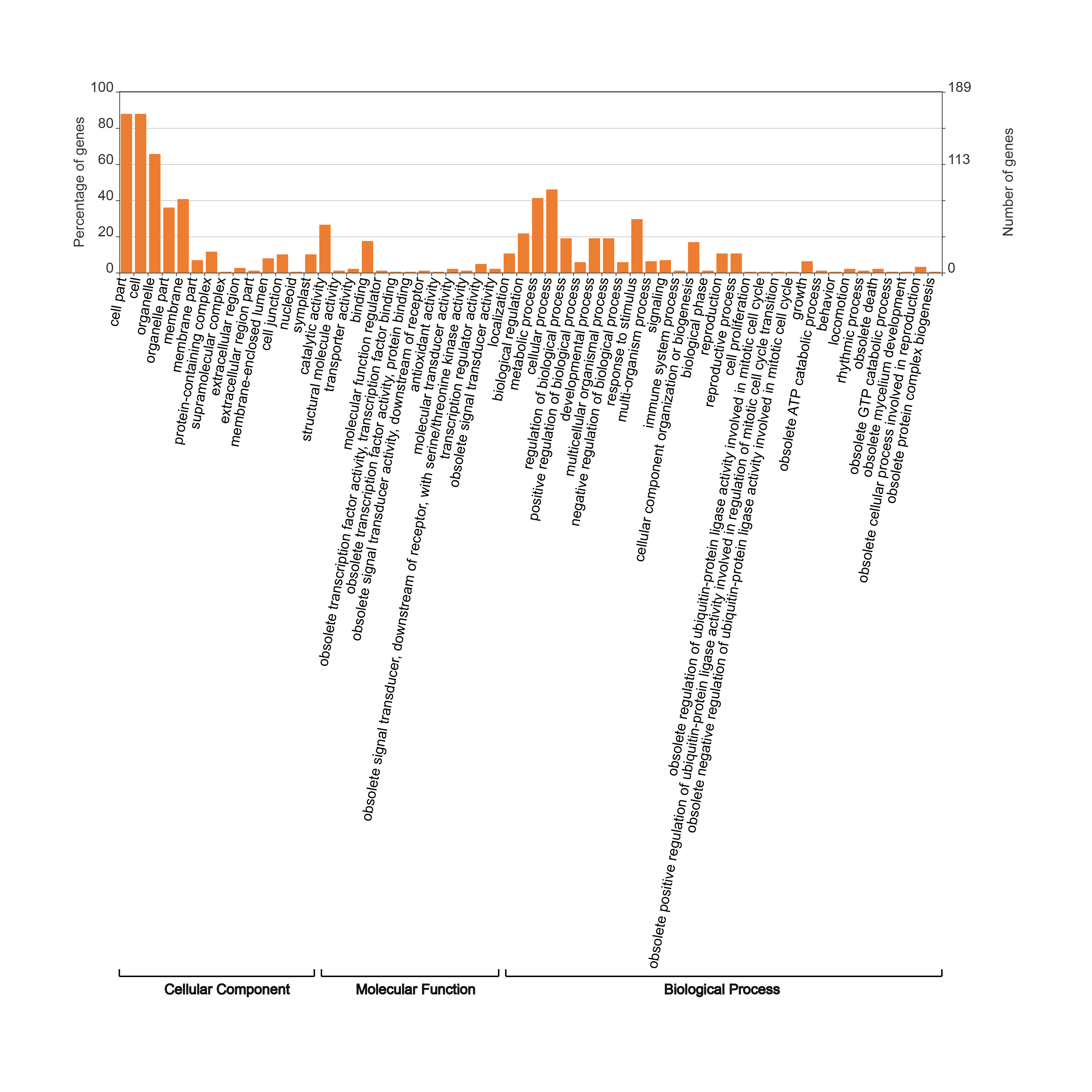


**Figure S2-H** ﻿GO enrichment analysis of ELD-C genes in stems of *B. napus.*


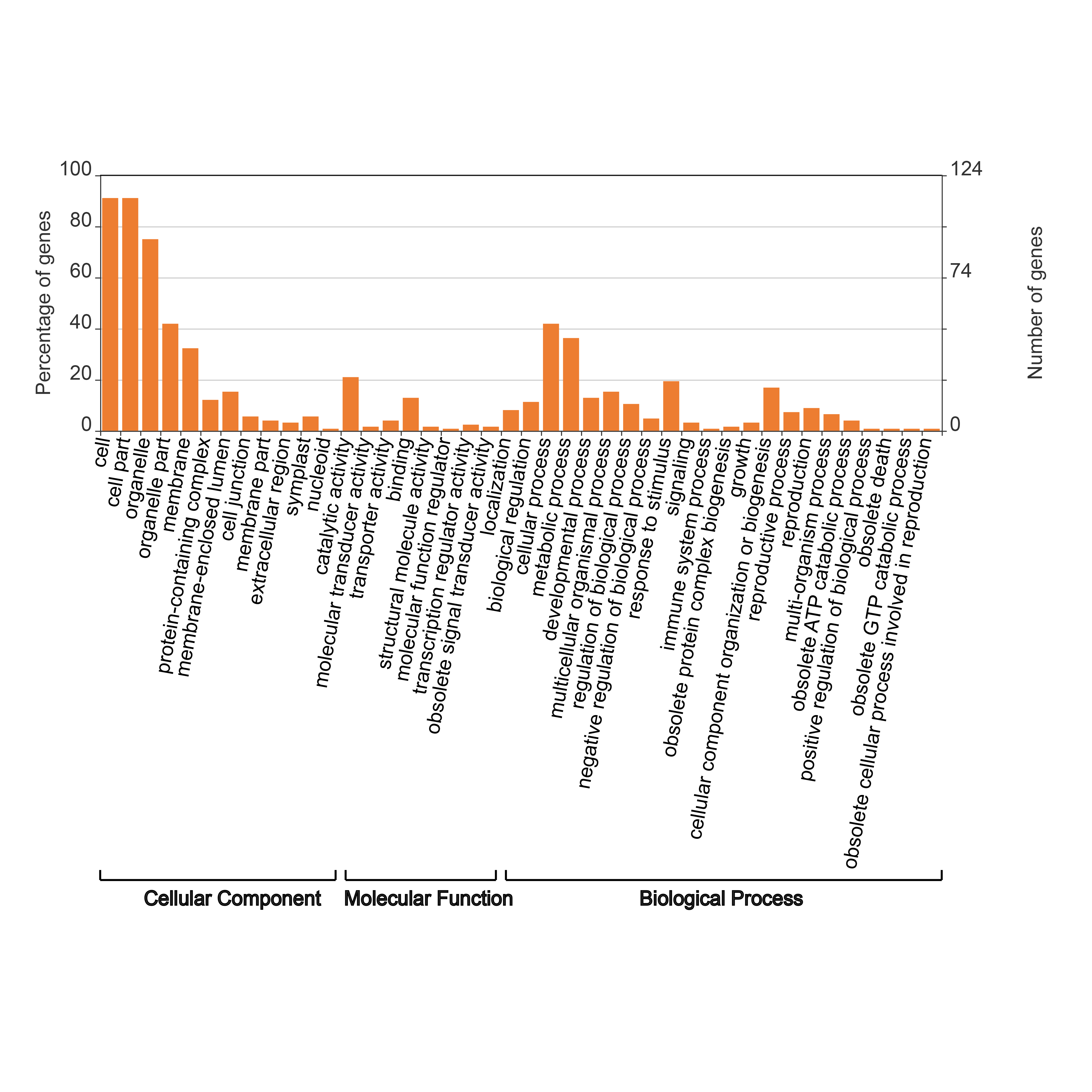

Supplement: Supplementary file 2 — Additional file 2 : Figure S2. GO enrichment analysis of ELD genes in four tissues of B. napus. [file 12864_2020_6747_MOESM2_ESM.docx]
