# Supplementary material for: Homoeolog expression bias and expression level dominance (ELD) in four tissues of natural allotetraploid Brassica napus
Source: BMC Genomics. 2020 Apr 29;21:330. doi: 10.1186/s12864-020-6747-1 (PMC7191788; doi:10.1186/s12864-020-6747-1)
Supplement: Supplementary file 6 — Additional file 6 : The perl script to identify orthologous gene pairs. [file 12864_2020_6747_MOESM6_ESM.rtf]

#!/usr/bin/env perldie "perl $0 blast1 blast2 >besthit.blast \n" unless @ARGV==2;my $in1 = shift;my $in2 = shift;my %h1 = readIN($in1);my %h2 = readIN($in2);foreach my $id (sort keys %h1){        my $id2 = $h1{$id}[0];        if(exists $h2{$id2} && $h2{$id2}[0] eq $id){                print "$h1{$id}[1]\n";        }}sub readIN{        my $in = shift;        my %hash;        open IN, $in ||die $!;        while(<IN>){                chomp;                my @t = split;                if(!exists $hash{$t[0]}){                        $hash{$t[0]}[0]=$t[1];                        $hash{$t[0]}[1]=$_;                }        }        close IN;        return %hash;}
